# Machine Learning-Based Detection of Graphene Defects with Atomic Precision

**DOI:** 10.1007/s40820-020-00519-w

**Published:** 2020-09-07

**Authors:** Bowen Zheng, Grace X. Gu

**Affiliations:** Department of Mechanical Engineering, University of California, Berkeley, CA 94720 USA

**Keywords:** Machine learning, Graphene, Defects, Molecular dynamics, Nanomaterials

## Abstract

Defects in graphene can profoundly impact its extraordinary properties, ultimately influencing the performances of graphene-based nanodevices. Methods to detect defects with atomic resolution in graphene can be technically demanding and involve complex sample preparations. An alternative approach is to observe the thermal vibration properties of the graphene sheet, which reflects defect information but in an implicit fashion. Machine learning, an emerging data-driven approach that offers solutions to learning hidden patterns from complex data, has been extensively applied in material design and discovery problems. In this paper, we propose a machine learning-based approach to detect graphene defects by discovering the hidden correlation between defect locations and thermal vibration features. Two prediction strategies are developed: an atom-based method which constructs data by atom indices, and a domain-based method which constructs data by domain discretization. Results show that while the atom-based method is capable of detecting a single-atom vacancy, the domain-based method can detect an unknown number of multiple vacancies up to atomic precision. Both methods can achieve approximately a 90% prediction accuracy on the reserved data for testing, indicating a promising extrapolation into unseen future graphene configurations. The proposed strategy offers promising solutions for the non-destructive evaluation of nanomaterials and accelerates new material discoveries.

**Electronic supplementary material:**

The online version of this article (10.1007/s40820-020-00519-w) contains supplementary material, which is available to authorized users.

## Highlights

- A machine learning-based approach is developed to predict the unknown defect locations by thermal vibration topographies of graphene sheets.
- Two prediction strategies are developed: an atom-based method which constructs data by atom indices, and a domain-based method which constructs data by domain discretization.
- Our machine learning model can achieve approximately a 90% prediction accuracy on the reserved data for testing, indicating a promising extrapolation into unseen future graphene configurations.

**Electronic supplementary material:**

The online version of this article (10.1007/s40820-020-00519-w) contains supplementary material, which is available to authorized users.

## Graphical abstract

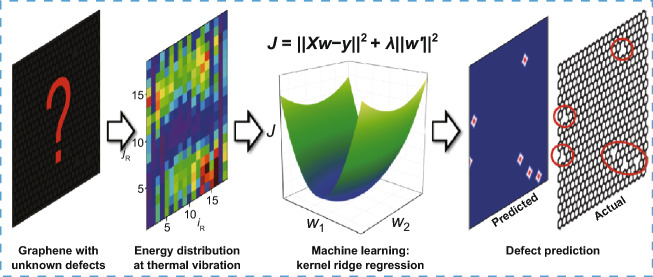

## Introduction

Graphene, due to its extraordinary electrical [1–3], thermal [4–6], and mechanical [7–10] properties, has been widely used as building blocks in high-performance nanoelectromechanical systems (NEMS) [11, 12], stretchable electronics [13, 14], supercapacitors [15, 16], among others. However, during the growth and processing of graphene the existence of defects is almost inevitable, which can compromise the expected performances of graphene-based nanodevices. Much research has been underway to understand the effect of defects on specific graphene properties [17–27]. Given the defect information such as size, location, and density, the properties of a defected graphene can be evaluated. Nevertheless, obtaining detailed defect information at atomic resolution is a difficult task. Ideally, defects such as vacancies can be discovered by examining the atomic structure of a graphene sheet. Despite some experimental successes using high-resolution transmission electron microscopy (TEM) [28–30], it remains technically challenging and involves complex sample preparation procedures to obtain an image of graphene at an atomic resolution. As a result, a method to reliably detect unknown graphene defects without using atomic-resolution probes is appealing.

Compared to elliptical holes and cracks which can be shed light on using traditional fracture mechanics, randomly distributed atomic vacancies have a much more implicit but not necessarily less profound impact on the mechanical properties of graphene. Emerging machine learning approaches offer solutions for learning patterns from complex data and have been extensively applied in material design and discovery problems [31–40]. The power of machine learning-based approaches can be fully utilized with a rational selection of features. In this problem, because the defect location is a local feature (instead of a global feature), data need to be constructed with observations possessing local information. Collective properties such as strength or strain to failure may not be suitable here, because defects at different locations can produce the same result, making these defect locations indistinguishable [18]. One of the simplest observations with local features is the thermal vibration at room temperature with all edges of the graphene sheet clamped, which does not require specific actuations or precise environment controls. Previous research has investigated the effect of defects on the vibrational properties of graphene via various technical approaches such as molecular dynamics (MD) simulation [41–43], continuum elasticity theory [42, 44, 45], and Monte Carlo-based finite element method [46]. The local amplitudes of thermal vibrations can be affected when surrounded by defects, because the absence of atoms changes the local boundary conditions of mini-oscillators. Experimentally, to obtain a vibration topography that has a lower resolution than atomic resolution is less taxing than obtaining an image of atomic structures. Low-amplitude mechanical vibrations of graphene can be readily imaged using a scanning force microscope [47] or an interferometry [12].

In this study, we propose a strategy to detect unknown defects in single-layer graphene sheets using machine learning to overcome the complicated relationship between thermal vibration topographies and defect locations. Trained by tens of thousands of thermal vibration topographies calculated by MD simulations, our machine learning model is used to predict defect locations. From predicting a single-atom vacancy to predicting an unknown number of vacancies with an arbitrary distribution, a kernel ridge regression model addresses problem by progressively building up the model complexity while maintaining the computational cost. Finally, an optimal model with the best prediction capability can be obtained by an extensive hyperparameter tuning. The proposed data-driven defect detection approach may contribute to the non-destructive evaluation of a broad variety of 2D materials and accelerate new material discoveries.

## Methods

### Molecular Dynamics Simulation

**Fig. 1:**
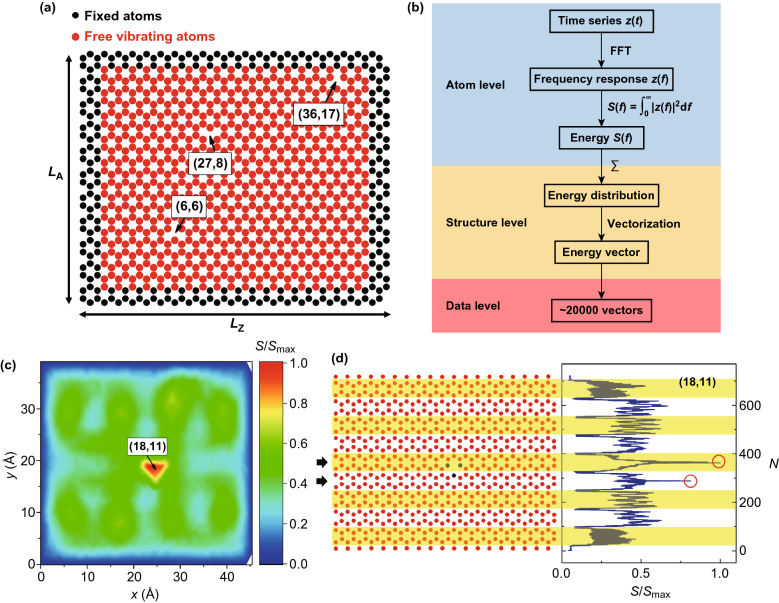
Descriptions of graphene sheets with vacancies and the procedure of data preparation. **a** Schematic of the defected graphene sheet and vacancy indexing, where a graphene sheet containing vacancies (6,6), (27,8), and (36,17) is used as an example. **b** Route of data preparation. **c** 2D energy distribution of graphene sheet with a vacancy (18,11), fixed atoms on the edges are not included in the contour plot. **d** 1D energy vector compressed from the 2D energy distribution and its correlation with the original graphene lattice

The thermal vibrations of single-layer graphene sheets are computed by MD simulations using the open-source code LAMMPS (Large-scale Atomic/Molecular Massively Parallel Simulator) [48]. An Adaptive Intermolecular Reactive Empirical Bond-Order (AIREBO) potential [49] is used to compute the interactions between pairs of carbon atoms in the graphene sheet. The AIREBO potential is composed of a REBO term to model the short-ranged interaction and a Lennard-Jones (LJ) term to model the long-ranged interaction, which can be formulated as Eq. (1):

1 $$E = \frac{1}{2}\mathop \sum \limits_{a} \mathop \sum \limits_{b \ne a} \left( {E_{ab}^{\text{REBO}} + E_{ab}^{\text{LJ}} } \right)$$

where *E* is the total potential energy accounting for all atomic interactions, and $E_{ab}^{\text{REBO}}$, $E_{ab}^{\text{LJ}}$ term the REBO potential and the LJ potential between atoms $a$ and $b$, respectively. In the REBO potential, two cutoff distances in the switching function control the bond-breaking behavior, which are by default 1.7 Å and 2.0 Å [49]. For simulations in this study, the value of the smaller cutoff distance is modified to 1.92 Å to accurately capture the mechanical behavior of graphene benchmarked by DFT calculations, a practice that has been validated by many previous studies [50–53]. The cutoff distance of the LJ term is 6.8 Å. Periodic boundary conditions are applied to two in-plane dimensions, and a fixed boundary condition is used in the orthogonal out-of-plane dimension. The box size is $D_{x} \times D_{y} \times D_{z}$ = 70 Å × 70 Å × 25 Å, where $D_{x}$, $D_{y}$, $D_{z}$ are the lengths of the box in $x$, $y$, $z$ directions, respectively. The integration time step is 1 femtosecond. An ensemble of random velocity at $T = 300\, {\text{K}}$ is generated throughout the graphene sheet. Graphene sheets are firstly relaxed in the canonical (NVT) ensemble at $T = 300 \;{\text{K}}$ for 10 ps. Then, the simulation is run in the isothermal–isobaric (NPT) ensemble with the Nose–Hoover thermostat [54] at the same temperature for 30 ps for graphene sheets to vibrate. The sampling frequency of atom displacement is 20 THz. The size of vacancy-containing graphene sheets is $L_{\text{Z}}$ = 52.1 Å by $L_{\text{A}}$ = 44.8 Å, where $L_{\text{Z}}$ and $L_{\text{A}}$ denote the zigzag and the armchair dimensions, respectively. The graphene sheet consists of 966 atoms when no defect is present. To enforce the boundary conditions, a 3-atom-wide stripe on each edge is set fixed by eliminating all degrees of freedom of the associated atoms, while the rest of the graphene sheet, composed of 38 rows and 19 columns of atoms, is free to vibrate. This boundary control resembles the experimental setup in Ref. [47], where the graphene sheet is clamped and suspended to vibrate with no substrate involved. Simulation temperature is chosen as the room temperature $T = 300\; {\text{K}}$, which requires the least temperature control in a potential experimental setup and can produce a sufficient vibration intensity. A location in the graphene sheet is indexed as (*i*, *j*), where *i* and *j* represent the *i*th row and the *j*th column, respectively. A vacancy can be referred to by the index of the location where an atom is missing. The size and the boundary conditions of graphene sheets, and the strategy of location indexing are illustrated in Fig. 1a. The easily satisfied loading conditions make both numerical and experimental approaches promising. For a pristine graphene sheet of this size and subject to the same boundary conditions, the amplitude of vibration is ~ 0.3 Å, which agrees well with quantitative results in Ref. [55]. The distribution of atom out-of-plane displacement during the thermal vibration of a pristine graphene sheet is provided in Fig. S1, where the graphene sheet is non-planar while vibrating.

### Data Preparation for Machine Learning

Training and testing data for machine learning implementations are prepared and organized into the following three levels: atom level, structure level, and data level. On the atom level, the time series of the out-of-plane displacement $z\left( t \right)$ of each atom is firstly computed. Then, a fast Fourier transformation is performed on $z\left( t \right)$ to obtain the frequency response $z\left( f \right)$. Next, the vibrational energy is calculated by $S\left( f \right) = \mathop \smallint \limits_{0}^{\infty } \left| {z\left( f \right)} \right|^{2} {\text{d}}f$, as a scalar to featurize each atom. Onto the structure level, an energy distribution throughout the graphene sheet is obtained by associating the energies of all atoms with their coordinates. Next, the 2D energy distribution is compressed to a 1D energy vector for the machine learning implementation. The energy vectors are based on atom indices, and the coordination information is suppressed. Finally, onto the data level, a total of near 20,000 energy vectors are prepared as the machine learning data and are assembled into a design matrix. The above procedure of data preparation is shown in the flowchart in Fig. 1b.

Among all presentations, the 2D energy distribution offers the best visualization. An example is provided in Fig. 1c, where the graphene sheet hosts a single-atom vacancy (18,11). As can be seen, the energy distribution is highly dependent on the location of vacancy: the vibrational energy tends to localize at defected regions. However, it is noteworthy that around the vacancy is not the global energy maximum, but a local maximum. The existence of vacancies creates additional local energy maxima off the energy distribution of pristine graphene, as is shown in the examples in Fig. S2. An energy vector compressed from the previous 2D energy distribution is illustrated in Fig. 1d, where the atom is indexed as $N = 19\left( {i - 1} \right) + j$. The energy vector reveals that one single-atom vacancy can produce not one but multiple characteristic spikes, which is not the most obvious in the 2D energy distribution. In addition, energy vectors, though less intuitive compared to energy distributions, offer another perspective and can be correlated with the original graphene structure. Considering each hexagonal ring of atoms as a unit, the graphene sheet can be divided into 9 rows of rings (RoRs) (the first and the last rows of atoms excluded). Each RoR is represented by a hump on the energy vector. Atoms surrounding the vacancy give rise to spikes on the humps that these atoms are associated with. For example, rows that are marked by two arrows in Fig. 1d are affected by the vacancy (18,11), hosting characteristic spikes. Atoms next to the fixed boundary exhibit low vibrational energy, as is the case for the first and the last row of atoms. Nevertheless, a vacancy in these atoms can still stimulate spikes, of which an example is provided in Fig. S3. This enables our machine learning approach to also predict vacancies next to the clamped edges.

## Results and Discussion

### Atom-Based Prediction of a Single-Atom Vacancy

The prediction of a single-atom vacancy, as the simplest case for the vacancy prediction, is developed first. The construction of energy vectors (featurized sample points) and label vectors is based on atom indices. The length of the energy vector is $722 - 1 = 721$ because of the one missing atom. All entries indexed after the missing atom need to be shifted accordingly. For example, if the 100th location corresponds to a vacancy, the energies of 101st to 722nd atoms are 100th to 721st entries of the energy vector. Label vectors are one-hot encoded, the length being the total number of possible atom locations. For example, if the *m*th location is a vacancy while others are occupied by atoms, the *m*th entry is 1 while other entries are 0’s. Despite that one-hot labels often work well with classification models, in this study they become infeasible due to the excessively many classes. For the scenario of a single-atom vacancy, the number of classes totals 722 (38 rows × 19 columns). For up to 10 vacancies, the number of classes grows to $$\sum\nolimits_{r = 1}^{10} {\left( {\begin{array}{*{20}c} {722} \\ r \\ \end{array} } \right)} \cong 1.01 \times 10^{22}$$, which goes far beyond realistic. Hence, a regressor is used to map energy vectors to one-hot labels. Kernel ridge regression model is selected to predict locations of vacancies, which enables us to progressively build up the model complexity without adding to the computational cost (the kernel trick). Hyperparameters include polynomial kernel degree $p$ and regularization parameter $\lambda$. Algorithmic details of kernel ridge regression are provided in Table 1.

**Table 1:** Machine learning algorithm details

Algorithm Kernel ridge regression	**a**
1: Normalize each energy vector ${\mathbf{x}}$ with its ${\mathcal{L}}_{2}$ norm, ${\mathbf{x}} \leftarrow {\mathbf{x}}/\|{\mathbf{x}}\|_{2}$.	
2: Center each energy vector ${\mathbf{x}}$ with the mean of all energy vectors ${\varvec{\upmu}} = \frac{1}{n}\mathop \sum \limits_{i = 1}^{n} {\mathbf{x}}_{i}$, ${\mathbf{x}} \leftarrow {\mathbf{x}} - {\varvec{\upmu}}$.	
3: Objective function $\text J\left( \text W \right) =\|{ \text {XW}} - \text Y\|^{2} + \uplambda \| \text W\|^{2}$, where $$X = \left[ {\begin{array}{*{20}c} {{\mathbf{x}}_{1} } & \cdots & {{\mathbf{x}}_{n} } \\ \end{array} } \right]^{\text{T}}$$ is the design matrix; $W$ is the weight matrix; $$Y = \left[ {\begin{array}{*{20}c} {{\mathbf{y}}_{1} } & \cdots & {{\mathbf{y}}_{n} } \\ \end{array} } \right]^{\text{T}}$$ is the label matrix.	
4: Normal equations $\left( {X^{\text{T}} X + \lambda I} \right)W = X^{\text{T}} Y$.	
5: Write $W$ as a linear transformation of sample points $W = X^{\text{T}} A$, where $A$ is the dual weight matrix.	
6: Objective function rewritten as $\text J\left( \text A \right) = \|{\text{XX}}^{\text{T}} \text A - \text Y\|^{2} + \uplambda \|\text X^{\text{T}} \text A\|^{2}$.	
7: Normal equations rewritten as $\left( {X^{\text{T}} X + \lambda I} \right)A = Y$.	
8: The polynomial kernel of degree $p$ is $k\left( {{\mathbf{q}}_{1} ,{\mathbf{q}}_{2} } \right) = \left( {{\mathbf{q}}_{1}^{\text{T}} {\mathbf{q}}_{2} + 1} \right)^{p}$.	
9: Construct kernel matrix $K$, $\forall i, j$, $K_{ij} \leftarrow k\left( {{\mathbf{x}}_{i} ,{\mathbf{x}}_{j} } \right)$.	
10: Solve $\left( {K + \lambda I} \right)A = Y$ for $A$.	
11: Predict labels for the design matrix of test data $$Z = \left[ {\begin{array}{*{20}c} {{\mathbf{z}}_{1} } & \ldots & {{\mathbf{z}}_{{n^{\prime}}} } \\ \end{array} } \right]^{\text{T}}$$ (${\mathbf{z}}_{i}$’s are normalized, centered testing energy vectors), $$\hat{Y} = \left[ {\begin{array}{*{20}c} {{\hat{\mathbf{y}}}_{1} } & \ldots & {{\hat{\mathbf{y}}}_{{n^{\prime}}} } \\ \end{array} } \right]^{\text{T}} = h\left( Z \right) = K^{\prime}A$$, where $K_{ij}^{'} = k\left( {{\mathbf{z}}_{i} ,{\mathbf{z}}_{j} } \right)$.	

Because there are in total 722 possible locations that are candidates to host a single-atom vacancy, to achieve an effective training process, all these possible locations need to be visited. Therefore, a total of 722 different configurations need to be simulated to survey all scenarios of the single-atom vacancy. Structured as the energy vector in Fig. 1d, results of 23 sets of 722 configurations (722 × 23 = 16,606 energy vectors in total) are prepared as data. The only difference between sets is the seed of random number generator of initial velocities, which ensures the data free from duplication or being a linear combination of any other data sets; 22 sets of data are used for training and validation, which, after random shuffling, are split into 80% for training and 20% for validation. An individual data set is set aside for testing. It is critical that the test data are not from shuffling and splitting from a large data set, but completely new, unseen data. A good performance on the test data can indicate promising extrapolation into future new sample points.

**Fig. 2:**
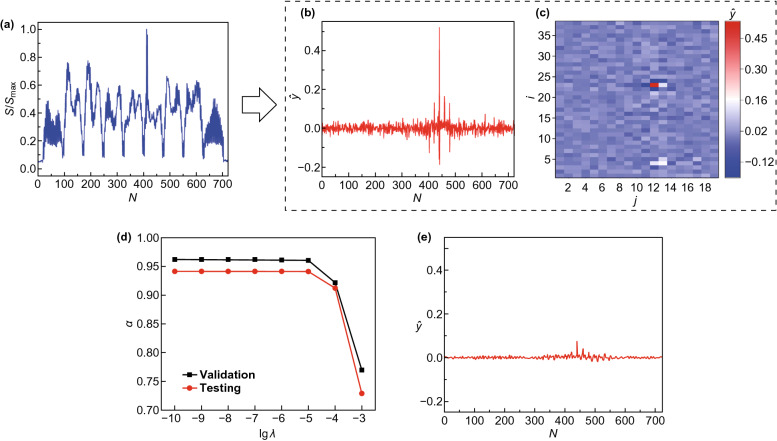
Results of the atom-based machine learning prediction for a single-atom vacancy. **a** An example of energy vector from the test data. **b** Predicted label vector. **c** 2D presentation of the predicted label vector. **d** Validation and testing accuracies as a function of regularization parameter $\lambda$. **e** Predicted label vector under a relatively strong regularization $\lambda = 10^{ - 3}$

To illustrate, an energy vector from the test set, normalized by its maximum entry value $S_{ \hbox{max} }$, is shown in Fig. 2a. The outstanding spike indicates that the vacancy potentially resides in its vicinity. The predicted label vector ${\hat{\mathbf{y}}}$ from the energy vector is shown in Fig. 2b, where $v = {\text{argmin}}_{{N \in \left\{ {1,2, \ldots ,722} \right\}}} \left| {\hat{y}_{N} - y_{N} } \right|$ is to be returned as the predicted vacancy location. To retrieve a better intuition from the prediction, ${\hat{\mathbf{y}}}$ is converted to 2D, as is shown in Fig. 2c where the predicted vacancy location stands out. Prediction accuracies $\alpha$ on the validation and the test data, as a function of regularization parameter $\lambda$, are shown in Fig. 2d. For $\lambda < 10^{ - 5}$, the validation accuracy is above 95% and the testing accuracy lies slightly below 95%, indicating a highly effective machine learning prediction. For a stronger regularization, for example, $\lambda = 10^{ - 3}$, the validation and the testing accuracies drop down to below 80% and 75%. Figure 2e shows the predicted label vector when $\lambda = 10^{ - 3}$. Although the noise level gets suppressed by a strong regularization, $\hat{y}_{v}$ becomes less preeminent, which explains the lowered prediction accuracies. Because both the validation and testing accuracies converge as $\lambda$ decreases, for this problem the machine learning model is not subject to high variance-related issues.

### Domain-Based Prediction of Multiple Vacancies with an Arbitrary Distribution

**Fig. 3:**
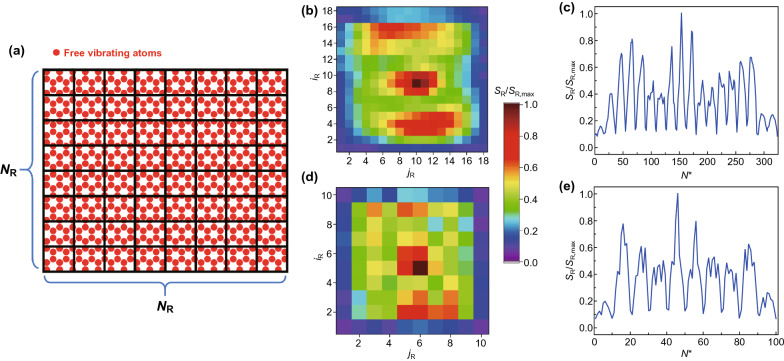
Domain discretization of a graphene sheet. **a** Schematic of an $N_{\text{R}}$-by-$N_{\text{R}}$ uniform domain discretization. **b** 2D energy distributions by 18-by-18 discretization converted from the atom-based energy vector in Fig. 2a. **c** 1D energy vector compressed from 18-by-18 discretization in (**b**). **d** 2D energy distribution of 10-by-10 discretization converted from the atom-based energy vector in Fig. 2a. **e** 1D energy vector compressed from 10-by-10 energy discretization in (**d**)

The atom-based method, despite a high prediction accuracy on the test set, becomes infeasible to predict multiple vacancies of an unknown quantity or density. This is because the length of energy vectors $722 - n_{\text{V}}$ is no longer a constant, where $n_{\text{V}}$ is the number of vacancies. More importantly, the atom-based method still requires counting atoms, which is not viable without an atomic-resolution probe. To circumvent this issue, an approach based on domain discretization is proposed, aiming to predict subdomains that contain one or more vacancies instead of the locations of missing atoms. The domain of free vibrating atoms is discretized into $N_{\text{R}}$-by-$N_{\text{R}}$ uniform subdomains, as is shown in Fig. 3a. Similar to the indexing strategy of the atom-based method, the index of a subdomain can be expressed as $N^{*} = \left( {i_{\text{R}} - 1} \right)N_{\text{R}} + j_{\text{R}}$, where $i_{\text{R}}$ and $j_{\text{R}}$ are the row index and the column index of a particular subdomain. Furthermore, when the size of the subdomains is substantially small, an atomic-resolution prediction can be approached. Notably, the domain-based method is computationally cheaper compared to the atom indexing-based method. For instance, for a graphene sheet with 722 freely vibrating atoms, the atom-based method renders each sample point $722 - n_{\text{V}}$ features. For domain-based method, the number of features is $N_{\text{R}}^{2}$ (for a 14-by-14 discretization, the number of features is 14^2^ = 196), thus achieving a dimensionality reduction by a multiple of $722/N_{\text{R}}^{2}$. Label vectors are one-hot encoded based on subdomains instead of atom indices, length being $N_{\text{R}}^{2}$: if the *s*th and *t*th subdomains contain a vacancy, the *s*th and *t*th entries of the energy vector are 1’s while the other entries are 0’s. Unlike the atom-based method, no index shift is involved.

As an immediate check for effectiveness, the same data used for the atom-based method are discretized and used to test the domain-based method: a good performance on the single-atom vacancy scenario must be achieved in order to proceed into predicting unknown multiple vacancies. To illustrate, the atom-based energy vector in Fig. 2a is converted to a domain-based energy vector. Figure 3b, c shows 2D and 1D presentations of the domain-based sample points with an 18-by-18 discretization, while Fig. 3d, e corresponds to a 10-by-10 discretization. Energy vectors of the domain-based method have less outstanding characteristic spikes compared to the atom-based counterpart, making defected regions almost indiscernible by an “eyeball” test and potentially adding to the difficulty of prediction.

**Fig. 4:**
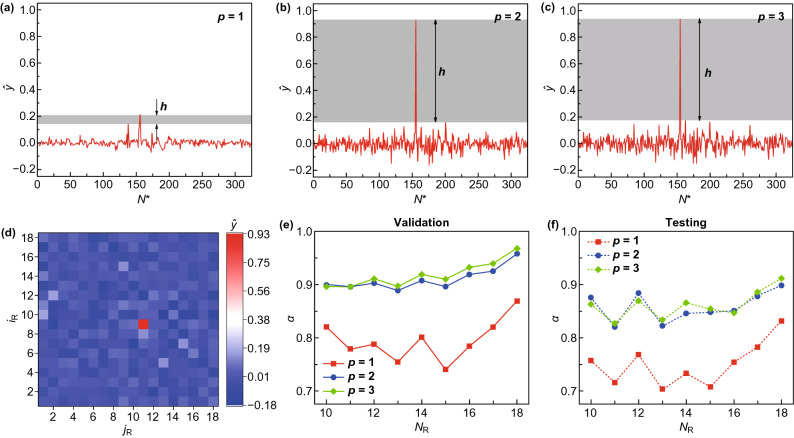
Machine learning predictions of a single-atom vacancy with domain discretization. **a**–**c** Predicted label vectors on an 18-by-18 discretization with kernel degrees 1, 2, and 3, respectively. The margin $h$ is illustrated by the gray areas. **d** 2D presentation of the predicted label vector of kernel degree 2. **e** Validation and **f** testing accuracies with kernel degrees $p \in \left\{ {1, 2, 3} \right\}$, as a function of $N_{\text{R}}$

To quantify the robustness of prediction, a margin is defined as $h = \min_{{N^{*} \in V}} \left| {\hat{y}_{{N^{*} }} } \right| - \max_{{N^{*} \notin V}} \left| {\hat{y}_{{N^{*} }} } \right|$, where $V$ is the set of indices of vacancy-containing subdomains. A large margin indicates that the machine learning model is less likely to confuse defected subdomains with pristine ones. $\lambda$ is kept small, set as $10^{ - 10}$. Input with the energy vector in Fig. 3c, the predicted label vector of machine learning model with a linear kernel is shown in Fig. 4a. Although $v = {\text{argmin}}_{{N^{*} \in \left\{ {1,2, \ldots ,N_{\text{R}}^{2} } \right\}}} \left| {\hat{y}_{{N^{*} }} - y_{{N^{*} }} } \right|$ can still correctly return the defected subdomain, the margin becomes particularly small and $\left| {\hat{y}_{v} - y_{v} } \right|$ becomes large, making predictions less reliable. To reduce the bias, polynomial kernels of higher degree are implemented. The predicted label vectors of quadratic and cubic kernels are shown in Figs. 4b, c. The margin is profoundly enlarged and $\left| {\hat{y}_{v} - y_{v} } \right|$ is sufficiently small for both cases, indicating a reliable prediction and a reduced bias. Little difference is observed between the predicted label vectors of quadratic and cubic kernels, indicating that a quadratic kernel already suffices to address the domain-based problem. A 2D presentation of the predicted label vector is provided in Fig. 4d, as a visualization with the best intuition. Validation and testing accuracies with kernel degrees $p \in \left\{ {1, 2, 3} \right\}$, as a function of $N_{\text{R}}$, are summarized in Figs. 4e, f. As can be seen, quadratic and cubic kernels, which have achieved accuracies over 90% on validation and over 80% on testing, are superior to a linear kernel. In addition, accuracies increase with larger $N_{\text{R}}$, i.e., finer discretization, despite some fluctuations in the testing accuracies. The effects of $\lambda$ on the validation and the testing accuracies are provided in Fig. S4.

**Fig. 5:**
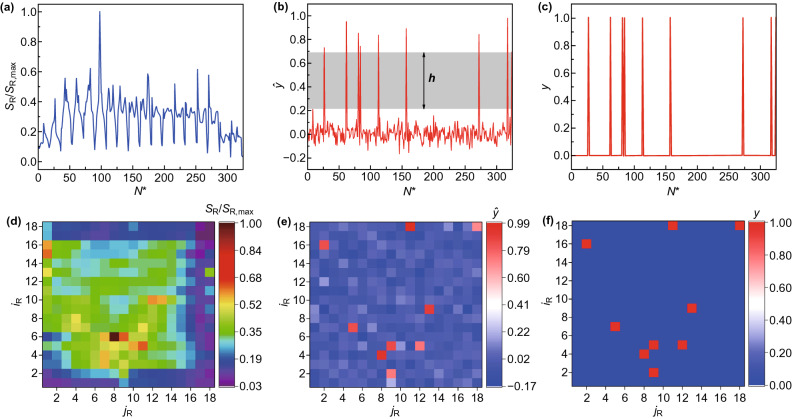
Machine learning predictions of multiple vacancies with domain discretization. **a** An example of energy vectors from the test data. **b** Predicted label vector by a quadratic kernel from the energy vector in **a**. **c** True label vector. **d**–**f** 2D presentations of (**a**–**c**), respectively

Having validated the domain-based method with predicting a single-atom vacancy, the model is used to predict locations of multiple vacancies with an arbitrary distribution. Data are prepared by the following way. The number of vacancies $n_{\text{v}}$ is a random integer from 1 to 10. Specifically, $n_{\text{v}} \sim {\mathcal{U}}\left( {1,10} \right)$, where ${\mathcal{U}}\left( \cdot \right)$ denotes a uniform distribution. The index of each vacancy is a pair of random integers corresponding to all possible atom locations, i.e., $i_{\text{R}} \sim {\mathcal{U}}\left( {1,38} \right)$ and $j_{\text{R}} \sim {\mathcal{U}}\left( {1,19} \right)$. This vacancy generation algorithm naturally does not rule out the existence of vacancy clusters, which free us from the issue of distinguishing between vacancy clusters and individual single-atom vacancies if the prediction is successful. This property is especially advantageous when the defect information is unknown a priori in an experimental setting. A total of 19,438 domain-based energy vectors are prepared by MD simulation, of which 80%, 10%, and 10% are used as training, validation, and test data, respectively. Each graphene configuration has a different seed of random number generator for the vacancy setup. Each simulation case also has a different seed for initial velocities. Once again, training and validation data are shuffled together and split into two sets, while the test data are not involved in any shuffling and splitting to be used as new data. Because of the unknown number of vacancies, returning ${\text{argmin}}_{{N^{*} \in \left\{ {1,2, \ldots ,N_{\text{R}}^{2} } \right\}}} \left| {\hat{y}_{{N^{*} }} - y_{{N^{*} }} } \right|$ or the indices of the $k$ smallest $\left| {\hat{y}_{{N^{*} }} - y_{{N^{*} }} } \right|$ as the predicted subdomain indices is no longer feasible. To this end, a threshold parameter $\tau$ is introduced and the set of indices of predicted defected subdomains can be obtained as $V = \left\{ {v: \hat{y}_{v} > \tau } \right\}$. $\lambda$ is set as $10^{ - 10}$. An example of energy vectors from the test data on an 18-by-18 discretization is shown in Fig. 5a. Multiple spikes are exhibited, but there is no intuition which of these spikes imply subdomains that contain vacancies. The predicted label vector by a quadratic kernel and the true label vector are shown in Figs. 5b, c, where a large margin is obtained. Given a threshold $\tau$ within the margin, the machine learning prediction returns 9 different subdomains that contain at least one vacancy, which are proved to be correct predictions by the true label vector in Fig. 5c. 2D presentations of the input data point, prediction label, and true label are shown in Figs. 5d–f, respectively, to offer a better intuition. For a sample point on the domain-based method, both 1D and 2D presentations have lost the ability to implicate locations of vacancies. However, the machine learning model can still discover the vacancies with high accuracy and reliability.

**Fig. 6:**
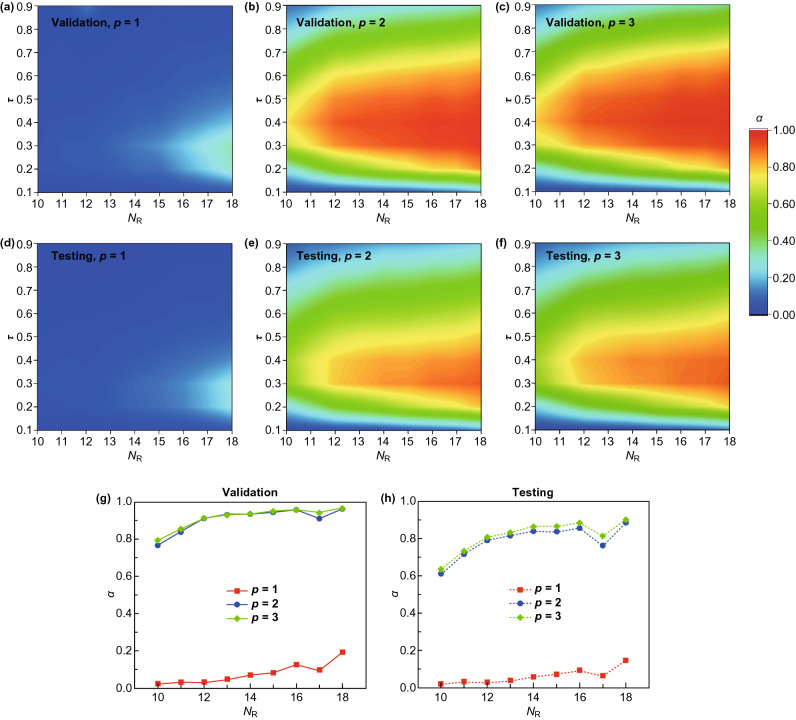
Parametric study and optimal performances of predicting multiple vacancies. **a**–**c** Testing accuracies of kernel degree $p \in \left\{ {1, 2, 3} \right\}$, as a function of $N_{\text{R}}$ and threshold $\tau$. **d**–**f** Validation accuracies of kernel degree $p \in \left\{ {1, 2, 3} \right\}$, as a function of $N_{\text{R}}$ and threshold $\tau$. **g** Validation and **h** testing accuracies as a function of $N_{\text{R}}$ with optimal choices of $\tau^{*}$

Validation accuracies of kernel degree $p \in \left\{ {1, 2, 3} \right\}$, as a function of $N_{\text{R}}$ and threshold $\tau$, are summarized in Figs. 6a–c, respectively. A linear kernel becomes incapable to predict vacancy locations, of which the best accuracy is below 40% and is only attainable when subdomain size is sufficiently small (for example, $N_{\text{R}} = 18$). However, for both quadratic and cubic kernels with an optimal $\tau^{*}$, validation accuracies above 80% can be achieved for $N_{\text{R}}$ values ranging from 10 to 18. As $N_{\text{R}}$ increases, validation accuracy increases and $\tau^{*}$ can be chosen within a broader range centered at near 0.4. Testing accuracies of kernel degree $p \in \left\{ {1, 2, 3} \right\}$, as a function of $N_{\text{R}}$ and threshold $\tau$, are shown in Figs. 6d–f, respectively. Trends in general resemble validation accuracies, but with a lower magnitude overall. Finally, prediction accuracies on validation and testing with optimal threshold values $\tau^{*}$ are summarized in Figs. 6g, h. As $N_{\text{R}}$ increases, the validation accuracy approaches 100% and the testing accuracy approaches 90%, suggesting a potent performance of locating multiple unknown vacancies in graphene sheets. The effects of $\lambda$ on the validation and the testing accuracies with $\tau = 0.4$ are provided in Fig. S5.

Both the atom-based and the domain-based methods can predict the locations of unknown vacancies with high accuracy. However, the latter is in general advantageous for multiple reasons. First, the domain-based method does not require an atomic-resolution probe, while the atom-based method does. Second, the domain-based method can predict an unknown number of vacancies, which makes it a more natural way to approach the problem. On the contrary, the atom-based method can only predict the vacancies of a known number, which poses an outstanding limit. Last but not least, the domain-based method enjoys cheaper computational cost and thus a faster training speed, due to the dimensionality reduction by discretization. Despite the fact that in order to achieve an over 90% prediction accuracy, the domain-based method requires at least a quadratic kernel while the atom-based method only needs a linear kernel, the kernel trick ensures that the computational costs of kernels of different degrees are generally equal. These advantages make the domain-based method more practical than the atom-based method for applications of interest. In an experimental setting, graphene samples can be fabricated by mechanical exfoliation following Ref. [47], which are relatively free of contamination such as oxygen-containing functional groups. For graphene sheets contaminated by foreign functional groups, based on the presented method these functional groups can be treated as defects and can be potentially distinguished from atomic vacancies. Also, it is suggested that the contamination layer can be removed by a high temperature cleaning process in a H_2_/Ar atmosphere, enabling measurements of the properties of contamination-free graphene sheets [56].

## Conclusions

In closing, we have provided a machine learning-based approach to predict locations of unknown vacancies in graphene. Thermal vibration properties at room temperature are used to featurize graphene sheets, which is shown to be effective to reveal the local vacancy information. Two prediction strategies are developed, an atom-based method which constructs data by atom indices, and a domain-based method which constructs data by domain discretization. Both strategies are based on a kernel ridge regression, which allows us to progressively build up model complexity while maintaining the computational cost. While the atom-based method is capable of predicting a single-atom vacancy, the domain-based method can predict an unknown number of multiple vacancies with high accuracy. Both methods can achieve approximately a 90% prediction accuracy on reserved test data, indicating a good extrapolation into unseen new graphene configuration. A dimensionality reduction is also achieved by domain discretization. The proposed machine learning-based approach shows a prediction capability beyond analytical and numerical modeling and can be further enhanced by the improvement in quality and speed of data generation. This strategy may also shed light on predicting defects of a broader variety, for instance, interstitials, dislocations, grain boundaries, among others. In the present study, graphene sheets are uniformed discretized. Non-uniform discretizations (for example, discretizations with gradients) or subdomains of irregular shapes are interesting studies for future work. Future endeavors also include the development and optimization of more complex discretization strategies, as well as predicting vacancies in multi-layer graphene sheets and other 2D materials.

## Electronic supplementary material

Below is the link to the electronic supplementary material.

Supplementary material 1 (PDF 425 kb)
