# Supplementary material for: Machine Learning-Based Detection of Graphene Defects with Atomic Precision
Source: Nanomicro Lett. 2020 Sep 7;12:181. doi: 10.1007/s40820-020-00519-w (PMC7770819; doi:10.1007/s40820-020-00519-w)
Supplement: Supplementary file 1 — Supplementary material 1 (PDF 425 kb) [file 40820_2020_519_MOESM1_ESM.pdf]

Supporting Information for

Machine Learning-Based Detection of Graphene Defects with Atomic Precision

Bowen Zheng[1], Grace X. Gu[1, *]

[1]Department of Mechanical Engineering, University of California, Berkeley, CA 94720, USA

*Corresponding author. E-mail: ggu@berkeley.edu (Grace X. Gu)

Supplementary Figures

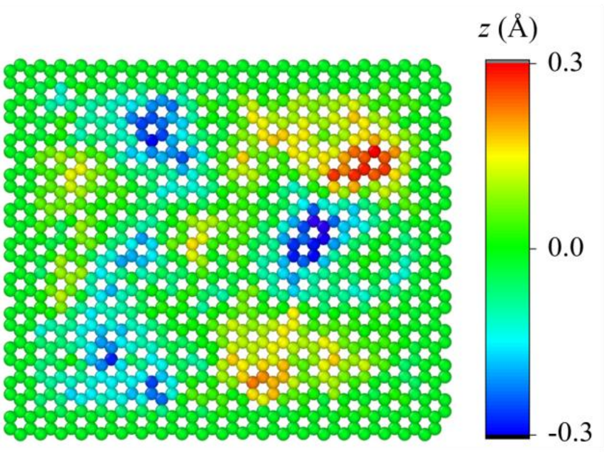

Fig. S1 Distribution of atom out-of-plane displacement during the thermal vibration of a pristine graphene sheet

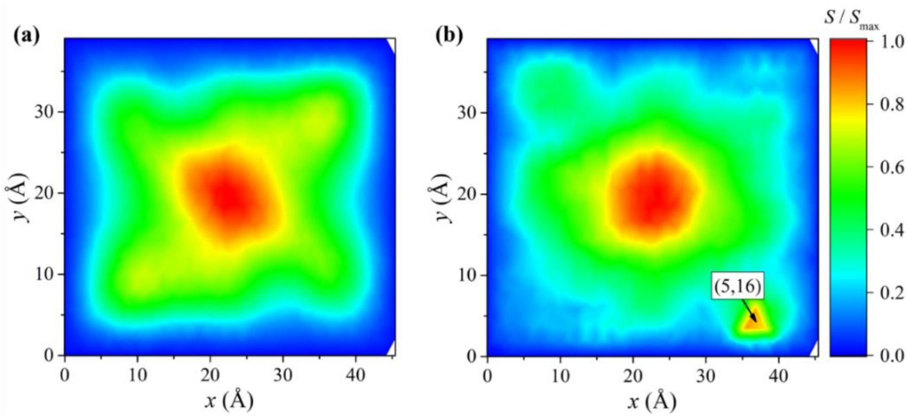

Fig. S2 2D energy distributions of (**a**) a pristine graphene sheet and (**b**) a graphene sheet with a vacancy (5, 16)

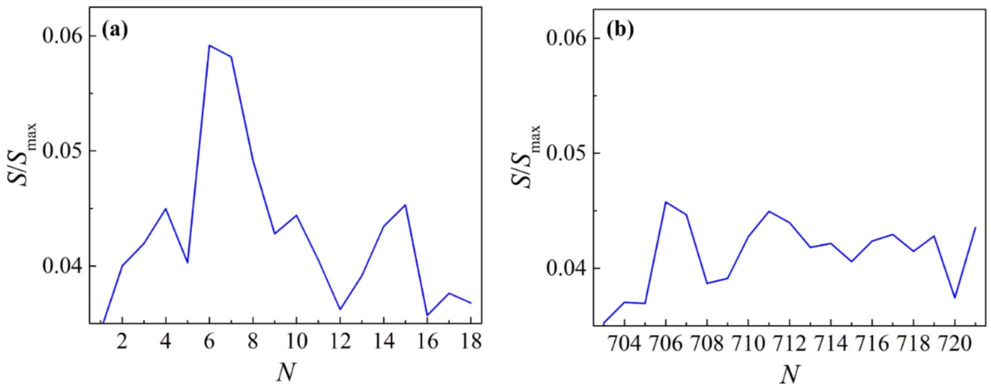

Fig. S3 Portions of energy vector corresponding to (**a**) the first row and (**b**) the last row of atoms. The graphene sheet here contains a single vacancy at (1,10), which lies on the first row of atoms

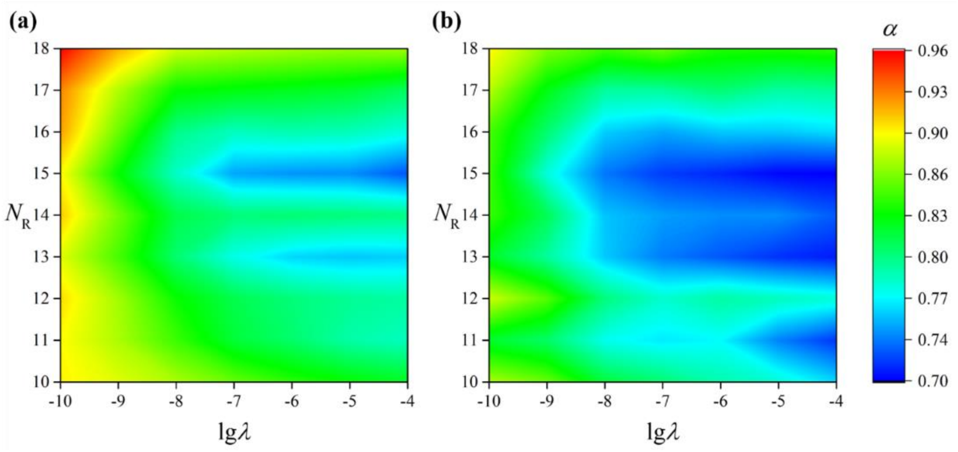

Fig. S4 Effect of regularization on predicting a single-atom vacancy by the domain-based method. (**a**) Validation and (**b**) testing accuracies as a function of N_{R} and λ

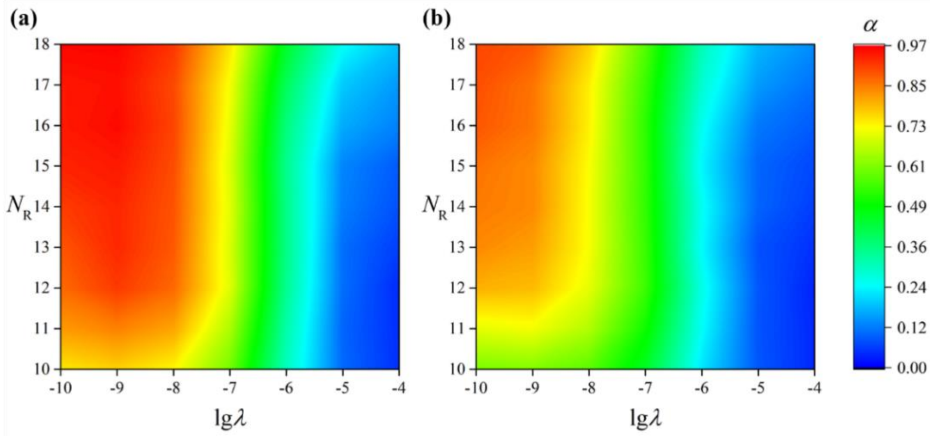

Fig. S5 Effect of regularization on predicting multiple vacancies by the domain-based method. (**a**) Validation and (**b**) testing accuracies as a function of N_{R} and λ, with $\tau = 0.4$
